# Determinants of incident atherosclerotic cardiovascular disease events among individuals with type 2 diabetic microvascular complications in the UK: a prospective cohort study

**DOI:** 10.1186/s13098-023-01152-4

**Published:** 2023-08-29

**Authors:** Yaxin Wang, Gabriella Bulloch, Yu Huang, Yingying Liang, Zijing Du, Guanrong Wu, Ying Fang, Yijun Hu, Xianwen Shang, Zhuoting Zhu, Xiayin Zhang, Xiaohong Yang, Honghua Yu

**Affiliations:** 1 Guangdong Eye Institute, Department of Ophthalmology, Guangdong Provincial People’s Hospital (Guangdong Academy of Medical Sciences), Southern Medical University, Guangzhou, China; 2 Centre for Eye Research Australia, Royal Victorian Eye and Ear Hospital, East Melbourne, VIC Australia; 3 Guangdong Cardiovascular Institute, Guangdong Provincial People’s Hospital (Guangdong Academy of Medical Sciences), Southern Medical University, Guangzhou, China; 4 Guangdong Provincial Key Laboratory of Artificial Intelligence in Medical Image Analysis and Application, Guangzhou, China

**Keywords:** Type 2 diabetes, Microvascular complication, Atherosclerotic cardiovascular disease, High-density lipoprotein cholesterol, Lipoprotein(a)

## Abstract

**Objective:**

To evaluate the association of atherosclerotic cardiovascular disease (ASCVD) risk factors with incident ASCVD events among type 2 diabetes (T2D) individuals with microvascular complications.

**Methods:**

We included T2D participants with only microvascular complications from the UK Biobank cohort at baseline (2006–2010). Multivariable-adjusted Cox proportional hazards models were used to study the association between ASCVD risk factors with adjudicated incident ASCVD in T2D participants with only microvascular complications. A restricted cubic spline approach was employed to evaluate potential nonlinear associations between ASCVD risk factors and ASCVD.

**Results:**

We studied 4,129 T2D individuals with microvascular complications at baseline. Over a median follow-up of 11.7 years, a total of 1,180 cases of incident ASCVD were documented, of which 1,040 were CHD, 100 were stroke, and 40 were both CHD and stroke events. After multivariable-adjustment, high-density lipoprotein cholesterol (HDL-C) level was linearly associated with a decreased risk of incident ASCVD [hazard ratio (HR): 0.49, 95% Confidence interval (CI): 0.32–0.75, P_linear_ = 0.011] and each 10 nmol/L increase of lipoprotein(a) [Lp(a)] level (HR: 1.02, 95% CI: 1.00-1.04, P_linear_ = 0.012) was linearly associated with an increased risk of incident ASCVD in T2D participants with only microvascular complications.

**Conclusion:**

HDL-C levels and Lp(a) levels (per 10 nmol/L) showed an independent linear relation with ASCVD risk among T2D individuals with only microvascular complications at long-term follow-up.

**Supplementary Information:**

The online version contains supplementary material available at 10.1186/s13098-023-01152-4.

## Introduction

Atherosclerotic cardiovascular disease (ASCVD) is a major macrovascular complication of diabetes [1]. It remains the principal cause of mortality among patients with type 2 diabetes (T2D) [2, 3]. To combat this critical health concern, the American College of Cardiology/American Heart Association (ACC/AHA) guidelines have strongly recommended lifestyle modification and medical control of ASCVD risk factors, aiming to minimize the incidence of ASCVD events [4]. Despite these recommendations, there remains a knowledge gap concerning the role of relevant ASCVD risk factors in patients with a history of T2D microvascular complications. Addressing this gap is essential for developing targeted preventive strategies and improving cardiovascular outcomes in this population.

Observational studies among those with T2D have repeatedly demonstrated that a series of unhealthy lifestyles and the burden of glycemia could exacerbate the potential risks of ASCVD [5–7]. The significance of hemoglobin A1c (HbA1c) as a predictor of cardiovascular disease and total mortality was evident in a study of 18,334 persons with T2D, where a linear relationship between HbA1c and macrovascular disease was observed, rather than a J-shaped [8]. Additionally, the pivotal role of low-density lipoprotein cholesterol (LDL-C) in atherosclerosis has long been recognized. This is particularly relevant in diabetes, as LDL-C is more atherogenic even without overtly increased LDL concentration [9]. Moreover, Elevated triglycerides, low high-density lipoprotein cholesterol (HDL-C), and higher concentrations of lipoprotein (a) [Lp(a)] also play significant roles in persons with diabetes developing ASCVD. Multiple risk factors influence the development of ASCVD, and interactions between these factors may further elevate the risk. In patients with T2D, the burden of smoking seems to be greater in women than men for coronary heart disease (CHD) morbidity, and the risk of fatal coronary events in women is 50% higher than in men [10]. An observational study in China, including 91,354 adults, found a significant interaction between diabetes and HDL-C concentrations on cardiovascular risk [11].

There is increasing evidence that microvascular complications have been consistently linked to the development of ASCVD in T2D patients [12–14]. Diabetic microvascular complications mainly contain diabetic retinopathy (DR) and diabetic kidney disease (DKD). DR is a leading cause of blindness and affects nearly one-third of adults with diabetes, [15] while DKD is the principal cause of end-stage renal failure in Western societies [16]. Importantly, DR and DKD are major risk factors for developing macrovascular complications such as ASCVD in patients with T2D [14, 17]. For individuals with microvascular complications *versus* none in T2D, the multivariable-adjusted hazard ratios for the cardiovascular events were 1.32–1.99 [13, 18]. These findings suggest that T2D patients with microvascular complications are higher-risk individuals for ASCVD than T2D only. Hence, we hypothesized that active management of ASCVD risk factors may help mitigate the progression from microvascular complications to ASCVD and related mortality in T2D patients. However, to validate this hypothesis, it is crucial to investigate the association between ASCVD risk factors and incident ASCVD events among T2D individuals with only microvascular complications during long-term follow-up.

In the present study, we evaluate whether common ASCVD risk factors, including cigarette smoking, hypertension, hyperlipidemia, family history of ASCVD, central obesity, lipid and inflammatory markers, serum vitamin D, and serum gamma-glutamyltransferase (GGT) are independently associated with incident ASCVD events among T2D participants with only microvascular complications over long-term follow-up in the UK mainly Caucasian.

## Materials and methods

### Study population of the UK Biobank

UK Biobank is an observational, population-based, prospective cohort study that recruited over 500,000 participants aged 40–69 years at baseline across 22 assessment centers throughout the United Kingdom. Information about the UK biobank data collection is available online (https://www.ukbiobank.ac.uk/), but in brief, baseline visits consisted of physical measurements, touchscreen questionnaires, and verbal interviews. In addition, blood, saliva, and urine samples were collected. Information about medical events was identified through hospital admission records and death registers.

The UK Biobank was conducted with ethics approval granted by the National Information Governance Board for Health and Social Care and the NHS North West Multicenter Research Ethics Committee (reference 11/NW/0382). All participants provided informed consent at the baseline assessment. The study was conducted adhering to the tenets of the Declaration of Helsinki and conducted under the UK Biobank application number 86091.

### Population for T2D with only microvascular complications

**Fig. 1:**
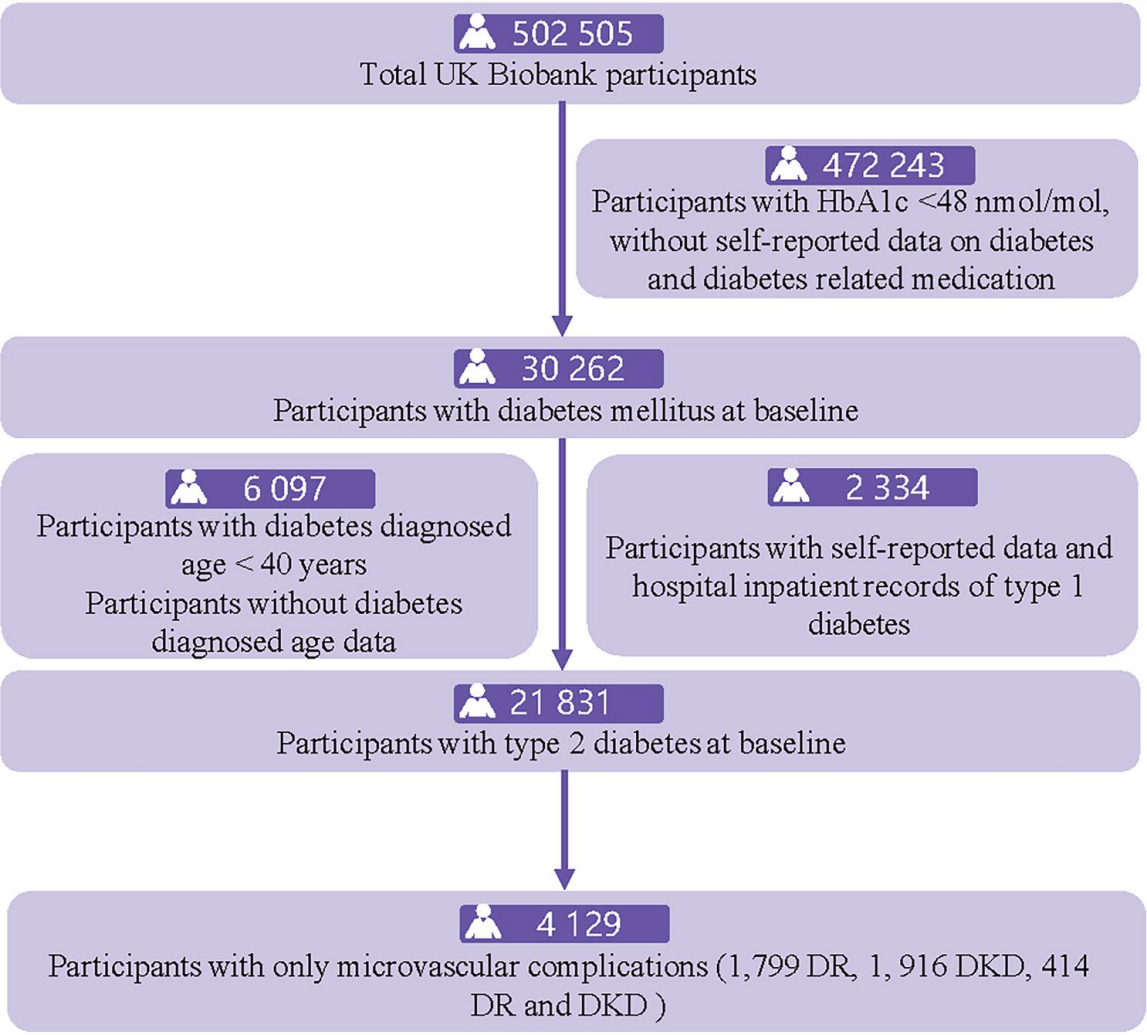
Flowchart for population selection for type 2 diabetes with microvascular complications from the UK Biobank

Diabetes was identified through linkage to “diabetes diagnosed by doctor”, HbA1c ≥ 48mmol/mol, and/or use of insulin and other diabetes-related medication at baseline. After excluding type 1 diabetes and diabetes diagnosed age < 40 years old, we obtained the participants with T2D (Fig. 1). Duration of diabetes was calculated based on the difference between baseline age and age at diagnosis of diabetes. Diabetes-related medication was recorded in insulin use, oral diabetes medication, or both.

T2D with only microvascular complications was defined as T2D participants with DR and/or DKD. The hospital inpatient records and the national death register data were defined using the codes for the International Classification of Diseases (ICD). DR was identified if self-reported, or hospital inpatient records made reference to DR, and about 10% of DR was identified by ophthalmologists using fundus photographs. DKD was identified through self-reported data, hospital inpatient records, and glomerular filtration rate (GFR) < 60 mL/min/1.73m^2^. The GFR calculation was based on serum cystatin C level, age, and sex.

### Ascertainment of incident ASCVD at follow-up

ASCVD cases in the UK Biobank study were identified through linkage to participants’ self-reported data, record linkage to hospital admissions data, and the national death register. Incident ASCVD was defined as the first occurrence of CHD events [myocardial infarction (ICD-10 codes I21–I23), resuscitated cardiac arrest (ICD-10 codes I46.0), and fatal CHD (ICD-10 codes I20–I25)] or stroke events [fatal and non-fatal events (ICD-10 codes I60–I64)]. The follow-up time was calculated from the date of baseline assessment and censored at the date of incident ASCVD events, date of death, date of loss to follow-up, or the end of follow-up (28th April 2021), whichever came first.

### Assessments of ASCVD risk factors and other covariates

Between 2006 and 2010, information pertaining to lifestyle factors (i.e., smoking, physical activity), comorbidities (i.e., hypertension, hyperlipidemia), medication (i.e., diabetes-related medication), and family history was collected using a touchscreen questionnaire during recruitment. Waist circumference was measured using the Wessex nonstretchable sprung tape. Central obesity was defined by a waist circumference of 88 cm or greater for females and 102 cm or greater for males. Smoking status was identified through linkage to “smoking status” and recorded as never or former/current cigarette smoking. Hypertension was identified through self-reported data, systolic blood pressure ≥ 130 mmHg or diastolic blood pressure ≥ 80 mmHg, or anti-hypertensive medication. Hyperlipidemia was identified through self-reported data, cholesterol ≥ 6.21 mmol/L, or taking statins and other anti-hyperlipidemia medication. HbA1c was divided into two groups based on the median HbA1c concentration.

Biomarker measures were performed using enzymatic (for triglycerides, high sensitivity C-reactive protein (CRP), and Lp(a), enzyme immuno-inhibition (for HDL-C), enzymatic rate (for GGT), or enzymatic selective protection (for LDL-C) methodology on the Beckman Coulter AU5800 platform. Serum 25-hydroxy vitamin D concentration was measured using the LIAISON XL 25(OH)D assay (DiaSorin, Stillwater, USA).

Other covariates included age, gender, ethnicity (recorded as white and others), education (recorded as graduate or professional school, and none), Townsend deprivation index (an area-based proxy measure for socioeconomic status), physical activity level (recorded as above moderate/vigorous/walking recommendation or not), which were collected during the initial assessment visit. UK biobank variables used in this paper were described in Supplementary Table 1.

### Statistical analysis

Baseline continuous variables were reported using mean ± standard deviation (SD), while categorical variables were summarized as count (percentage). Student t-tests were used to test the difference in continuous variables and χ^2^ test in categorical variables.

Unadjusted incidence rates of events reported as the number of events per 1000-person-years among those with the presence versus absence of ASCVD risk factors were calculated. Risk ratios (RRs) and 95% CIs were estimated. Multivariable Cox proportional hazards models were applied to examine the association between ASCVD risk factors and incident ASCVD for participants with T2D microvascular complications at baseline. Hazard ratios (HRs) and 95% CIs were estimated. We first adjusted models for age, gender, ethnicity, education, Townsend deprivation index, physical activity, duration of diabetes, and diabetes-related medication (model 1); Model 2 included adjustments for model 1 plus HbA1c, cigarette smoking, hypertension, hyperlipidemia, family history of ASCVD, central obesity, serum HDL-C, serum triglycerides, serum LDL-C, serum Lp(a), serum vitamin D, and serum GGT. The variance inflation factor (VIF) was calculated to investigate the collinearity among variables.

The association between HDL-C, Lp(a) levels and the incidence of ASCVD, CHD, and stroke outcomes was evaluated on a continuous scale with restricted cubic spline curves based on Cox proportional hazards models. Analyses were adjusted for multiple variables with 5 knots located at the 5th, 25th, 50th, 75th and 95th percentiles for HDL-C and Lp(a). If a test for nonlinearity was insignificant, we performed a linearity test, comparing a model containing the linear term with a model containing covariates. In sensitivity analyses, the association between ASCVD risk factors and incident ASCVD was examined by excluding individuals who developed ASCVD in the first 2 years of follow-up.

Data analyses were conducted using Stata version 16.0 (StataCorp, College Station, TX) and R (version 4.2.0, R Foundation for Statistical Computing, www.R-project.org, Vienna, Austria). A two-tailed P < 0.05 was considered significant.

## Results

The study population consisted of 4,129 T2D participants with only microvascular complications with a mean (± SD) age of 61.9 (± 6.0) years and 41.1% female (Fig. 1). Of them, 1,799 were DR, 1,916 were DKD, and 414 were both DR and DKD. The mean (± SD) duration of diabetes was 8.6 (± 8.4) years. 8.8% of T2D participants with only microvascular complications were treated with insulin, and 74.8% with oral diabetes medication. The median HbA1c concentration was 50.6 nmol/mol. Compared to those who did not develop ASCVD, T2D participants with only microvascular complications who did were significantly older (62.7 vs. 61.6 years), performed fewer activities (65.4% vs. 72.9%), more likely to use insulin (10.8% vs. 8.0%), with longer duration of diabetes (9.7 vs. 8.2 years), lower HDL-C level (1.1 vs. 1.2 mmol/L), higher triglycerides level (2.3 vs. 2.2 mmol/L), lower LDL-C level (2.6 vs. 2.7 mmol/L), higher Lp(a) level (47.2 vs. 41.6 mmol/L), higher HbA1c level (54.2 vs. 53.0 mmol/mol), lower vitamin D level (39.1 vs. 41.6 nmol/L); all P < 0.05 (Table 1).

**Table 1:** Baseline characteristic of the study population among those with type 2 diabetic microvascular complications, stratified by the development of incident atherosclerotic cardiovascular disease

Baseline characteristics	Incident ASCVD		*P* value
	**No (n = 2,949)**	**Yes (n = 1,180)**	
Age, years	61.6 ± 6.2	62.7 ± 5.6	**< 0.001**
Gender			**< 0.001**
Female	1,333 (45.2%)	363 (30.8%)	
Male	1,616 (54.8%)	817 (69.2%)	
Ethnicity			0.671
White	2,480 (84.1%)	986 (83.6%)	
Non-White	469 (15.9%)	194 (16.4%)	
Education			0.135
None	2,380 (80.7%)	976 (82.7%)	
Graduate or professional school	569 (19.3%)	204 (17.3%)	
Townsend deprivation index	0.1 ± 3.5	0.3 ± 3.5	0.126
Above moderate/vigorous/walking recommendation			**< 0.001**
No	591 (27.1%)	300 (34.6%)	
Yes	1,592 (72.9%)	567 (65.4%)	
Diabetes-related medication			
Insulin use	187 (8.0%)	103 (10.8%)	**0.007**
Oral diabetes medication	1,791 (76.7%)	671 (70.1%)	**0.022**
Insulin use + Oral diabetes medication	356 (15.3%)	188 (19.1%)	**0.003**
Duration of diabetes, year	8.2 ± 8.2	9.7 ± 8.8	**< 0.001**
Cigarette smoking status			**< 0.001**
Never	1,428 (48.8%)	473 (40.6%)	
Former/Current	1,498 (51.2%)	691 (59.4%)	
Hypertension			0.614
No	267 (9.1%)	101 (8.6%)	
Yes	2,682 (90.9%)	1,079 (91.4%)	
Hyperlipidemia			**0.002**
No	553 (18.8%)	174 (14.7%)	
Yes	2,396 (81.2%)	1,006 (85.3%)	
Family history of ASCVD			**< 0.001**
No	1,209 (41.0%)	408 (34.6%)	
Yes	1,740 (59.0%)	772 (65.4%)	
Central obesity			0.314
No	800 (27.1%)	302 (26.0%)	
Yes	2,149 (72.9%)	878 (74.0%)	
HDL-C, mmol/L	1.2 ± 0.3	1.1 ± 0.3	**< 0.001**
Triglycerides, mmol/L	2.2 ± 1.2	2.3 ± 1.3	**0.003**
LDL-C, mmol/L	2.7 ± 0.8	2.6 ± 0.7	**0.010**
Lipoprotein(a), nmol/L	41.6 ± 47.6	47.2 ± 48.9	**0.004**
HbA1c, nmol/mol	53.0 ± 13.9	54.2 ± 13.7	**0.014**
CPR, mg/L	4.2 ± 6.1	4.5 ± 6.5	0.101
Vitamin D, nmol/L	41.6 ± 19.9	39.1 ± 19.8	**< 0.001**
Gamma-glutamyltransferase, U/L	52.0 ± 68.1	55.5 ± 57.4	0.125

Continuous variables are summarized as mean ± standard deviation. Categorical variables are summarized as count (percentage). Abbreviations: ASCVD (atherosclerotic cardiovascular disease); HDL-C (high-density lipoprotein cholesterol); LDL-C (low-density lipoprotein cholesterol); HbA1c (hemoglobin A1c); CRP (C-reactive protein). **BOLDED** items are significant statistically significant (P < 0.05)

Over a median follow-up of 11.7 years, 1,180 ASCVD events occurred among those with only microvascular complications at baseline, of which 1,040 were CHD, 100 were stroke, and 40 were both CHD and stroke events. ASCVD event rates were higher among former/current cigarette smokers than never-smokers (29/1000 versus 22/1000 person-years, RR: 1.31, 95% CI: 1.22–2.75). ASCVD event rates were 31/1000 person-years among those without increased GGT and 23/1000 person-years among those with increased GGT level (RR: 0.73, 95% CI: 0.65–0.83). The unadjusted event rates of ASCVD were ≤ 30 per 1000-person-years for most risk factors, except lower GGT (30.8) (Supplementary Table 2).

**Fig. 2:**
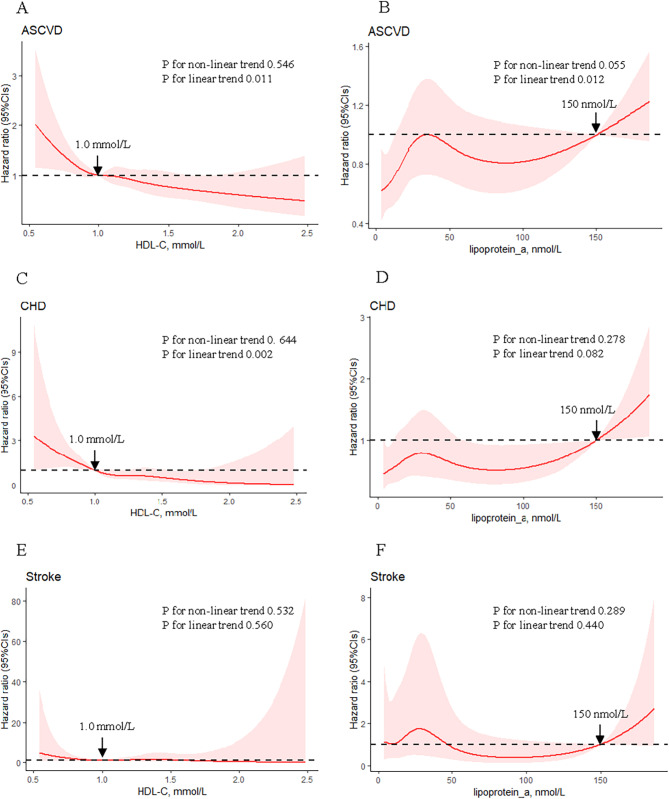
Association between serum high density lipoprotein cholesterol (HDL-C), lipoprotein(a) (Lp(a)) and outcomes, allowing for non-linear effects. Association between serum HDL-C and risks of atherosclerotic cardiovascular disease (**A**), coronary heart disease (**C**), and stroke (**E**) in T2D participants with only microvascular complications. The reference HDL-C (with a hazard ratio fixed as 1.0) was 1.0 mmol/L (**A**, **C**, **E**). Association between serum Lp(a) and risks of atherosclerotic cardiovascular disease (**B**), coronary heart disease (**D**), and stroke (**F**) in T2D participants with only microvascular complications. The reference Lp(a) (with a hazard ratio fixed as 1.0) was 150 nmol/L (**B**, **D**, **F**)

Hazard ratios (95% CI) for the association of cardiovascular risk factors and incident ASCVD among T2D participants with only microvascular complications were presented in Table 2 (VIF < 4 in all models). After multivariable-adjustment, risk factors that remained significantly associated with ASCVD were lower HDL-C level (HR: 0.49, 95% CI: 0.32–0.75, P = 0.001) and higher Lp(a) level (HR: 1.02, 95% CI: 1.00-1.04, P = 0.019, per 10 nmol/L increment). Multivariable-adjusted restricted cubic spline analyses suggested a J-shape association between levels of HDL-C on a continuous scale and risk of ASCVD (P_linear_ = 0.011, Fig. 2A). The significant linear relationship remained for Lp(a) and incident ASCVD (P_linear_ = 0.012, Fig. 2B). A sensitivity analysis among 4,106 participants who did not develop ASCVD in the first 2 years of follow-up showed that the association between ASCVD risk factors and ASCVD was consistent with the main findings (Supplementary Table 3).

**Table 2:** Multivariable-adjusted hazard ratios (95% Confidence interval) for the association of ASCVD risk factors and incident atherosclerotic cardiovascular disease among those with type 2 diabetic microvascular complications at baseline

ASCVD risk factors	ASCVD Model 1	ASCVD Model 2
Cigarette smoking-Former/Current	**1.23 (1.04–1.45)**	1.20 (0.99–1.47)
Hypertension	0.97 (0.73–1.30)	0.95 (0.66–1.37)
Hyperlipidemia	1.24 (0.98–1.57)	1.13 (0.85–1.51)
Family history of ASCVD	**1.20 (1.02–1.41)**	1.20 (0.98–1.46)
Central Obesity	**1.20 (1.00-1.44)**	1.09 (0.87–1.37)
Triglycerides, per mmol/L	1.07 (1.01–1.14)	0.99 (0.90–1.09)
HDL-C, per mmol/L	**0.59 (0.43–0.80)**	**0.49 (0.32–0.75)**
LDL-C, per mmol/L	1.04 (0.93–1.16)	1.07 (0.91–1.25)
Lipoprotein(a), per 10 mmol/L	**1.02 (1.00-1.04)**	**1.02 (1.00-1.04)**
CRP, per mg/L	**1.02 (1.01–1.03)**	1.01 (0.99–1.02)
Vitamin D, per nmol/L	**0.99 (0.99-1.00)**	1.00 (0.99-1.00)
Gamma-glutamyltransferase, per 10 U/L	1.01 (1.00-1.02)	1.02 (1.00-1.03)

Model1 is adjusted for age, gender, ethnicity, education, Townsend deprivation index, physical activity, duration of diabetes, diabetes-related medication, and hemoglobin A1c; Model2 is adjusted for age, gender, ethnicity, education, Townsend deprivation index, physical activity, duration of diabetes, diabetes-related medication, hemoglobin A1c, and all listed risk factors. Abbreviations: ASCVD (atherosclerotic cardiovascular disease); HDL-C (high-density lipoprotein cholesterol); LDL-C (low-density lipoprotein cholesterol); CRP (C-reactive protein). **BOLDED** items are significant statistically significant (P < 0.05)

The significant linear relationship remained for HDL-C and incident CHD in T2D participants with only microvascular complications (HR: 0.14, 95% CI: 0.05–0.38, P < 0.001; P_linear_ = 0.002, Fig. 2C). No association was indicated for Lp(a) and incident CHD (P_non−linear_ =0.278, Fig. 2D). In addition, for HDL-C and incident stroke, no association was indicated (P_non−linear_ = 0.532, Fig. 2E). No association was indicated for Lp(a) and incident stroke (P_non−linear_ = 0.289, Fig. 2F). The association between ASCVD risk factors and incident CHD, stroke was shown in Supplementary Table 4.

In sex-stratified analyses, higher Lp(a) level (HR: 1.02, 95% CI: 1.00-1.04, P = 0.028) was significantly associated with ASCVD among female only. Lower HDL-C (HR: 0.42, 95% CI: 0.25–0.70, P = 0.001) level was significantly associated with ASCVD among male only. There was no significant interaction between Lp(a) and HDL-C level with sex in the multivariable-adjusted Cox regression model (P_for interaction_ > 0.05, Supplementary Table 5).

For HbA1c and incident ASCVD, no association was indicated after multivariable adjustment in T2D participants with only microvascular complications (Supplementary Table 6). In the HbA1c subgroup, lower HDL-C level was significantly associated with ASCVD among patients with HbA1c > 50.6nmol/mol (HR: 0.52, 95% CI: 0.31–0.87, P = 0.012). There was no significant interaction between all ASCVD risk factors (P_for interaction_ > 0.05) with HbA1c in the multivariable-adjusted Cox regression model except CRP level (P_for interaction_ = 0.009, Supplementary Table 7).

## Discussion

In this large prospective cohort study, we investigated the association between ASCVD risk factors and incident ASCVD events in T2D patients with only microvascular complications during a long-term follow-up. Several conclusions can be drawn from the present study. First, HDL-C and Lp(a) levels (per 10 nmol/L) showed an independent linear relationship with incident ASCVD among individuals with only microvascular complications at baseline. Second, a lower HDL-C level was significantly associated with CHD, while no risk factor showed a significant association with stroke. Third, in sex-stratified analyses, a higher Lp(a) level was significantly associated with ASCVD events among female only, whereas a lower HDL-C level was significantly associated with ASCVD events among male.

Our results revealed a significant association between lower serum HDL-C and an increased risk of incident ASCVD events in T2D patients with microvascular complications. This finding aligns with previous evidence that highlighted the essential role of HDL as a protective factor for cardiovascular health and vascular disease risk since the 1980s [19, 20]. The Emerging Risk Factors Collaboration conducted a study in 2009, analyzing data from 68 long-term prospective studies, and confirmed HDL-C’s independent and inverse association with ASCVD [19]. However, in contrast, a recent study reported a paradoxical association between high HDL-C levels and all-cause mortality [21]. Serum HDL-C plays a vital role in suppressing inflammation, oxidation, and thrombosis in the vascular system and has anti-atherogenic properties by promoting cholesterol efflux of intravascular fat deposits, thereby preventing the formation of fatty streaks [22]. Moreover, HDL-C removes cholesterol from foam cells, leading to the reversal of atherosclerotic plaque formation [23]. In diabetes individuals, dyslipidemia is a crucial feature of its diabetics. Recent research has shown that glycation can lead to reduced and dysfunctional HDL-C bioactivity, potentially diminishing its protective role in ASCVD [24]. Laboratory studies have demonstrated that glycated HDL-C from type 1 diabetics did not minimize cholesterol efflux capacity, irrespective of glycemic control [25]. While our analyses demonstrated that high HDL-C levels remained protective against ASCVD events in our study population, the emerging research suggesting limited bioavailability of HDL-C in people with diabetes warrants further investigation. Understanding the intricate relationship between HDL-C, diabetes, and ASCVD risk may offer valuable insights for developing targeted preventive strategies in this vulnerable population. In our sex-stratified analyses, we observed a significant association between lower HDL-C levels and ASCVD risk among male only, while no significant differences were found among female. This finding aligns with a previous study on HDL-C and stroke in the middle-aged and elderly population [26]. The lack of significance among female could be attributed to the possibility that elevated HDL-C may not always confer cardioprotective benefits, particularly in postmenopausal women [27].

In our analysis of ASCVD events, we observed a small but significant 2% increase in the risk of ASCVD in T2D patients with microvascular complications associated with each 10 nmol/L increment in serum Lp(a) levels. This finding aligns with previous epidemiological and Mendelian randomization studies, which have consistently identified Lp(a) as an independent causal factor for ASCVD [28–30]. Lp(a) is an atherogenic lipoprotein composed of an LDL–like moiety with one plasminogen-like apolipoprotein-A covalently bound to apolipoprotein B. Circulating levels of Lp(a) are primarily determined by hereditary modifications at the *LPA* gene locus, leading to its association with familial hyperlipidemia [31]. Interestingly, while several cohort studies have shown that low Lp(a) levels were associated with an increased risk of T2D, [32, 33] the risk of cardiovascular events in patients with diabetes appears to be positively associated with serum Lp(a) levels [34, 35]. A multicenter study highlighted that elevated Lp(a) levels were related to a significantly higher risk of cardiovascular events in stable coronary artery disease (CAD) patients with pre-diabetes or diabetes compared to those with normal glucose regulation [36]. Similarly, another study in Chinese patients demonstrated that elevated Lp(a) is independently associated with the presence and severity of CAD in T2D patients [37]. The risk observed in these studies was much higher than in the current study (HR: 1.56–3.47). One potential explanation for the lower risk magnitude observed in our study may lie in the characteristics of our reference cohort. Our study cohort already had microvascular complications in the past, unlike studies that compared healthy controls or diabetics without microvascular disease. Possibly due to the progressive nature of the disease in the individuals examined within this cohort, elevated serum Lp(a) did not confer the advanced risk of ASCVD at the same level of magnitude compared to non-diabetics or diabetics without microvascular involvement. Overall, our findings contribute to the growing body of evidence linking Lp(a) to ASCVD risk in T2D patients with microvascular complications, and they underscore the importance of considering the unique characteristics of patient cohorts when interpreting the impact of Lp(a) on cardiovascular outcomes.

Our study revealed a lack of association between HbA1c and incident ASCVD in the population with microvascular complications, which was inconsistent with previous study [8]. Several factors might explain this discrepancy. Firstly, a substantial proportion of the selected T2D population in our study was managed using insulin or diabetes-related medications, potentially influencing the baseline HbA1c levels. Secondly, we carefully adjusted for the duration of diabetes in our analysis. These factors might have contributed to the difference in the observed association between HbA1c and incident ASCVD compared to the previous study. Interestingly, our results highlight the potential significance of HDL-C and Lp(a) as more critical risk factors for incident ASCVD in T2D patients with microvascular complications than HbA1c. These findings suggest that lipid-related factors, rather than glycemic control measured by HbA1c, may substantially influence the cardiovascular risk profile in this population.

The implications of our findings are highly relevant to public health. With the global prevalence of diabetes in adults projected to rise from 6.4 to 7.7% by 2030 and juvenile diabetes becoming more common, an increasing number of individuals are living with diabetes long-term, putting them at heightened risk for micro- and macrovascular complications [38]. Our study observed high unadjusted incidence rates for ASCVD (> 19 per 1000 person-years) in the diabetic microvascular complications population. Given the significant associations we found with HDL-C and Lp(a), specific attention should be given to monitoring these abnormal serum markers in this at-risk population. Our study underscores the importance of understanding the associations between various risk factors and incident ASCVD events in individuals with microvascular complications. Additional clinical considerations for ASCVD prevention can be identified by shedding light on these associations, leading to more effective preventive strategies tailored to this high-risk population. Moreover, our findings suggest that targeted interventions to manage decreased HDL-C and increased Lp(a) levels may offer protective benefits to T2D patients with only microvascular complications, potentially reducing the incidence of ASCVD events in this population.

Strengths of this study include its large prospective cohort, long-term follow-up, comprehensive demographic characteristics, and biomarker availability. In addition, the longitudinal recording of disease and changes in serum markers allow for comprehensive analysis and adjustment for confounding variables. Some limitations should also be acknowledged. First, the definition of T2D might lead to misclassification of diabetes type. Second, the prevalence of microvascular complications at baseline was 22.5%, lower than that of previous studies (> 40% for DKD) [39, 40]. So, some cases with microvascular complications might not be captured at baseline. Furthermore, although we aimed to adjust for confounders as much as possible, some residual confounding may still exist. Lastly, participants in this study were recruited from the UK Biobank cohort, which is known as a relatively healthy cohort from a higher socioeconomic background and with a large proportion of Caucasian ethnicity. This could introduce selection bias and limit the generalizability to other population groups.

In summary, our study highlights the importance of controlling ASCVD events in T2D patients with only microvascular complications. Lower levels of HDL-C and higher levels of Lp(a) were independently associated with an increased risk of incident ASCVD events in this specific population during long-term follow-up. This study emphasizes the need for special attention to HDL-C and Lp(a) levels in controlling ASCVD events in T2D patients with microvascular complications.

### Electronic supplementary material

Below is the link to the electronic supplementary material.

Supplementary Material 1

## Data Availability

The UK Biobank data are available on the application to the UK Biobank (www.ukbiobank.ac.uk/).

## Abbreviations

ASCVD: Atherosclerotic Cardiovascular Disease
T2D: Type 2 Diabetes
HbA1c: Hemoglobin A1c
LDL-C: Low-density Lipoprotein Cholesterol
HDL-C: High-density Lipoprotein Cholesterol
Lp(a): Lipoprotein(a)
CHD: Coronary Heart Disease
DR: Diabetic Retinopathy
DKD: Diabetic Kidney Disease
GGT: Gamma-glutamyltransferase
ICD: International Classification of Diseases
GFR: Glomerular Filtration Rate
CRP: C-reactive Protein
CAD: Coronary Artery Disease
