# Supplementary material for: Determinants of incident atherosclerotic cardiovascular disease events among individuals with type 2 diabetic microvascular complications in the UK: a prospective cohort study
Source: Diabetol Metab Syndr. 2023 Aug 29;15:178. doi: 10.1186/s13098-023-01152-4 (PMC10463420; doi:10.1186/s13098-023-01152-4)
Supplement: Supplementary file 1 — Supplementary Material 1 [file 13098_2023_1152_MOESM1_ESM.docx]

**Supplementary material**

**Supplementary Table 1.** **UK Biobank showcase variables used in the paper.**

| **Measurements** | **Field ID** | **Time** | **Description** |
| --- | --- | --- | --- |
| **Diabetes** | 2443 | Baseline assessment | Doctor-diagnosed diabetes. Touchscreen question: "Has a doctor ever told you that you have diabetes?" |
|  | 20003 |  | The use of anti-hyperglycemic medications. |
|  | 6153  (code 3) |  | The use of insulin. |
|  | 30750 |  | Glycated hemoglobin level measured by HPLC analysis on a Bio-Rad VARIANT II Turbo (≥ 48 mmol/mol). |
| **Type 2 diabetes** (Type 2 diabetes + diabetes excluded type 1 diabetes and diabetes diagnosed age < 40 years) | 41270 (code E11-E119) | Baseline assessment | Hospital in-patient records with insulin-dependent diabetes as main or any secondary diagnoses based on the 10th edition of the WHO International Classification of Diseases (ICD-10). |
|  | 20002 (code 1223) |  | Self-reported type 2 diabetes. |
|  | 41270 (code E10-E109) |  | Hospital in-patient records with non-insulin-dependent diabetes as main or any secondary diagnoses based on the 10th edition of the WHO International Classification of Diseases (ICD-10). |
|  | 20002 (code 1222) |  | Self-reported type 1 diabetes. |
|  | 2976 |  | Age diabetes diagnosed (≥40 years). Question asked: "What was your age when the diabetes was first diagnosed?" |
| **Diabetic retinopathy** | 6148 (code 1) | Baseline assessment | Diabetic related eye disease. Question: "Has a doctor told you that you have any of the following problems with your eyes?" |
|  | 20002 (code 1276) |  | Self-reported diabetic retinopathy. |
|  | 41270 (code H360, E113) |  | Hospital in-patient records with diabetic retinopathy as the main or any secondary diagnosis based on the 10th edition of the WHO International Classification of Diseases (ICD-10). |
|  | 41271 (code 3620, 2504) |  | Hospital in-patient records with diabetic retinopathy as the main or any secondary diagnosis based on the 9th edition of the WHO International Classification of Diseases (ICD-9). |
| **Diabetic kidney disease** | 20002 (code 1607) | Baseline assessment | Self-reported diabetic kidney disease. |
|  | 20002 (code 1192, 1193, 1194, 1519) |  | Self-reported chronic kidney disease. |
|  | 41270 (code E112, N083) |  | Hospital in-patient records with diabetic kidney disease as the main or any secondary diagnosis based on the 10th edition of the WHO International Classification of Diseases (ICD-10). |
|  | 41271 (code 2503) |  | Hospital in-patient records with diabetic kidney disease as the main or any secondary diagnosis based on the 9th edition of the WHO International Classification of Diseases (ICD-9). |
|  | 30720 |  | Cystatin C. The glomerular filtration rate (GFR) calculation was based on serum cystatin C level, age, and sex. Diabetic kidney disease was identified through linkage to GFR <60 mL/min/1.73m2. |
| **Incident ASCVD events** | | | |
| **Incident coronary heart disease** | 41270 (code I20-I25, I460) | From the date of baseline assessment to the date of onset coronary heart disease/ stroke | Hospital in-patient records with coronary heart disease as the main or any secondary diagnosis based on the 10th edition of the WHO International Classification of Diseases (ICD-10). |
|  | 40001 (code I20-I25, I460) |  | National death register with coronary heart disease. Underlying/primary cause of death reported for the participant. Acquired from the central registry. |
| **Incident stroke** | 41270 (code I60-I64) |  | Hospital in-patient records with stroke as main or any secondary diagnoses based on the 10th edition of the WHO International Classification of Diseases (ICD-10). |
|  | 40001 (code I60-I64) |  | National death register with stroke. Underlying/primary cause of death reported for the participant. Acquired from the central registry. |
| **Risk factors** | | | |
| **Smoking status** | 20116 | Baseline assessment | This field summarizes the current/past smoking status of the participant. |
| **Hypertension** | 20002 (code 1065, 1072) | Baseline assessment | Self-reported hypertension. |
|  | 6153 (code 2) |  | Use of antihypertensive drugs. |
|  | 4080 |  | Average systolic blood pressure of at least 130mmHg. |
|  | 4079 |  | Average diastolic blood pressure of at least 80mmHg. |
| **Hyperlipidemia** | 20002 (code 1473) | Baseline assessment | Self-reported hyperlipidemia. |
|  | 6153 |  | The use of statins. |
|  | 20003 |  | The use of hyperlipidemia-related medication. |
|  | 30690 |  | Blood cholesterol level Measured by CHO-POD analysis on a Beckman Coulter AU5800 (≥ 6.21 mmol/L). |
| **Family history of atherosclerotic cardiovascular disease** | 20107  (code 12) | Baseline assessment | Illnesses of father. Touchscreen question "Has/did your father ever suffer from? (You can select more than one answer)". |
|  | 20110  (code 12) |  | Illnesses of mother. Touchscreen question "Has/did your mother ever suffer from? (You can select more than one answer)". |
|  | 20111  (code 12) |  | Illnesses of siblings. Touchscreen question: "Have any of your brothers or sisters suffered from any of the following diseases? (You can select more than one answer)". |
| **Central obesity** | 48 | Baseline assessment | Waist circumference. Central obesity is defined by a waist circumference of 88 cm or greater for females and 102 cm or greater for males. |
| **Triglycerides** | 30870 | Baseline assessment | Blood triglycerides level Measured by enzymatic methodology on a Beckman Coulter AU5800 (mmol/L). |
| **High-density lipoprotein cholesterol** | 30760 | Baseline assessment | Blood high-density lipoprotein cholesterol level Measured by enzyme immuno-inhibition methodology on a Beckman Coulter AU5800 (mmol/L). |
| **Low-density lipoprotein cholesterol** | 30780 | Baseline assessment | Blood low-density lipoprotein cholesterol level Measured by enzymatic selective protection methodology on a Beckman Coulter AU5800 (mmol/L). |
| **Lipoprotein(a)** | 30790 | Baseline assessment | Blood lipoprotein(a) level Measured by enzymatic methodology on a Beckman Coulter AU5800 (nmol/L). |
| **C-reactive protein** | 30710 | Baseline assessment | Blood C-reactive protein level Measured by enzymatic methodology on a Beckman Coulter AU5800 (mg/L). |
| **Vitamin D** | 30890 | Baseline assessment | Serum 25-hydroxy vitamin D concentration Measured by LIAISON XL 25(OH)D assay (nmol/L) |
| **Gamma- glutamyltransferase** | 30730 | Baseline assessment | Blood gamma- glutamyltransferase Measured by enzymatic rate methodology on a Beckman Coulter AU5800 (U/L). |
| **Demographic information** | | | |
| **Age** | 21003 | Baseline assessment | Refer to the age of the participant on the day they attended an Assessment Centre, year. |
| **Sex** | 31 | Baseline assessment | Sex of participant. |
| **Ethnic background** | 21000 | Baseline assess-ent | Recorded as white and non-white. |
| **Education attainment** | 6138 | Baseline assessment | Touchscreen question: "Which of the following qualifications do you have? (You can select more than one)". |
| **Townsend deprivation index** | 189 | Baseline assessment | Townsend deprivation index calculated immediately prior to participant joining the UK Biobank based on the preceding national census output areas. Each participant is assigned a score corresponding to the output area in which their postcode is located. |
| **Physical activity levels** | 22036 | Baseline assessment | Indicates whether a person met the 2017 UK Physical Activity Guidelines of 150 minutes of walking or moderate activity per week or 75 minutes of vigorous activity. |

**Supplementary Table 2.** Unadjusted incidence rates of atherosclerotic cardiovascular disease among those with type 2 diabetic microvascular complications at baseline (median follow-up time 11.7 years)

| ASCVD risk factors | ASCVD (per 1000-person years) | Risk ratios (RR) (95% confidence intervals (CI)) |
| --- | --- | --- |
| Cigarette smoking status |  |  |
| Never | 22.20 |  |
| Former/Current | 29.00 | 1.31 (1.16-1.47) |
| Hypertension |  |  |
| No | 25.73 |  |
| Yes | 25.89 | 1.01 (0.82-1.25) |
| Hyperlipidemia |  |  |
| No | 21.97 |  |
| Yes | 26.70 | 1.22 (1. 03-1.44) |
| Family history of ASCVD |  |  |
| No | 22.79 |  |
| Yes | 27.88 | 1.22 (1.08-1.38) |
| Central Obesity |  |  |
| No | 24.78 |  |
| Yes | 26.28 | 1.06 (0.93-1.21) |
| Decreased HDL-C |  |  |
| No | 24.49 |  |
| Yes | 27.53 | 1.12 (0.99-1.27) |
| Triglycerides ≥1.7 mmol/L |  |  |
| No | 24.93 |  |
| Yes | 26.73 | 1.07 (0.95-1.21) |
| LDL-C ≥ 4.9 mmol/L |  |  |
| No | 26.08 |  |
| Yes | 19.34 | 0.74 (0.32-1.47) |
| Lipoprotein(a) ≥ 125 nmol/L |  |  |
| No | 25.83 |  |
| Yes | 28.32 | 1.10 (0.88-1.36) |
| CRP ≥ 2 mg/L |  |  |
| No | 24.57 |  |
| Yes | 27.21 | 1.11 (0.98-1.25) |
| Vitamin D < 30 nmol/L |  |  |
| No | 24.40 |  |
| Yes | 29.42 | 1.21 (1.07-1.36) |
| Increased Gamma-glutamyltransferase |  |  |
| No | 30.84 |  |
| Yes | 22.62 | 0.73 (0.65-0.83) |

Abbreviations: ASCVD (atherosclerotic cardiovascular disease); HDL-C (high-density lipoprotein cholesterol); LDL-C (low-density lipoprotein cholesterol); CRP (C-reactive protein).

Decreased HDL-C is defined as ≤ 1.0 mmol/L for men or ≤ 1.3 mmol/L for women.

Increased gamma-glutamyltransferase is defined as ≥55 U/L for men or ≥38 U/L for women.

**Supplementary Table 3.** Multivariable-adjusted hazard ratios (95% confidence interval) for the association of ASCVD risk factors and incident atherosclerotic cardiovascular disease among those with type 2 diabetic microvascular complications by excluding individuals who developed ASCVD in the first 2 years of follow-up

| ASCVD risk factors | Multivariable-adjusted hazard ratios for ASCVD |
| --- | --- |
| Cigarette smoking-Former/Current | 1.20 (0.98-1.47) |
| Hypertension | 0.99 (0.68-1.44) |
| Hyperlipidemia | 1.08 (0.81-1.45) |
| Family history of ASCVD | **1.24 (1.01-1.51)** |
| Central Obesity | 1.08 (0.85-1.36) |
| Triglycerides, per mmol/L | 0.98 (0.89-1.08) |
| HDL-C, per mmol/L | **0.47 (0.30-0.72)** |
| LDL-C, per mmol/L | 1.03 (0.87-1.21) |
| Lipoprotein(a), per 10 mmol/L | **1.02 (1.00-1.04)** |
| CRP, per mg/L | 1.01 (0.99-1.02) |
| Vitamin D, per nmol/L | 1.00 (0.99-1.00) |
| Gamma-glutamyltransferase, per 10 U/L | 1.01 (0.99-1.03) |

The model is adjusted for age, gender, ethnicity, education, Townsend [deprivation index,](http://www.mendeley.com/research/townsend-deprivation-index/) physical activity, duration of diabetes, diabetes-related medication, hemoglobin A1c and all listed risk factors. Abbreviations: ASCVD (atherosclerotic cardiovascular disease); HDL-C (high-density lipoprotein cholesterol); LDL-C (low-density lipoprotein cholesterol); CRP (C-reactive protein).
**BOLDED** items are significant statistically significant (P < 0.05).

**Supplementary Table 4.** Multivariable-adjusted hazard ratios (95% confidence interval) for the association of ASCVD risk factors and incident coronary heart disease and stroke among those with type 2 diabetic microvascular complications at baseline

| ASCVD risk factors | Multivariable-adjusted hazard ratios for CHD | Multivariable-adjusted hazard ratios for stroke |
| --- | --- | --- |
| Cigarette smoking-Former/Current | 1.09 (0.71-1.66) | 0.73 (0.34-1.59) |
| Hypertension | 0.65 (0.33-1.28) | 0.92 (0.21-3.99) |
| Hyperlipidemia | 1.89 (0.91-3.94) | 0.83 (0.29-2.35) |
| Family history of ASCVD | 1.44 (0.93-2.22) | 0.88 (0.41-1.90) |
| Central Obesity | 0.94 (0.56-1.58) | 0.81 (0.31-2.12) |
| Triglycerides, per mmol/L | 0.89 (0.74-1.08) | 1.05 (0.77-1.43) |
| HDL-C, per mmol/L | **0.14 (0.05-0.38)** | 0.45 (0.09-2.41) |
| LDL-C, per mmol/L | 1.29 (0.93-1.79) | 1.21 (0.68-2.15) |
| Lipoprotein(a), per 10 mmol/L | 1.04 (1.00-1.08) | 0.98 (0.90-1.06) |
| CRP, per mg/L | 1.02 (1.00-1.05) | 0.99 (0.93-1.06) |
| Vitamin D, per nmol/L | **0.98 (0.96-0.99)** | 0.98 (0.95-1.00) |
| Gamma-glutamyltransferase, per 10 U/L | 1.00 (0.97-1.03) | 1.03 (0.99-1.07) |

The model is adjusted for age, gender, ethnicity, education, Townsend [deprivation index,](http://www.mendeley.com/research/townsend-deprivation-index/) physical activity, duration of diabetes, diabetes-related medication, hemoglobin A1c and all listed risk factors. Abbreviations: ASCVD (atherosclerotic cardiovascular disease); HDL-C (high-density lipoprotein cholesterol); LDL-C (low-density lipoprotein cholesterol); CRP (C-reactive protein).
**BOLDED** items are significant statistically significant (P < 0.05).

**Supplementary Table 5.** Multivariable-adjusted hazard ratios (95% confidence interval) for the association of ASCVD risk factors and incident atherosclerotic cardiovascular disease among those with type 2 diabetic microvascular complications stratified by gender at baseline

| ASCVD risk factors | Female | Male |
| --- | --- | --- |
| Cigarette smoking-Former/Current | 1.25 (0.83-1.88) | 1.18 (0.93-1.48) |
| Hypertension | **4.30 (1.01-18.4)** | 0.79 (0.54-1.15) |
| Hyperlipidemia | 0.96 (0.53-1.75) | 1.17 (0.83-1.64) |
| Family history of ASCVD | 1.11 (0.72-1.72) | 1.21 (0.96-1.51) |
| Central Obesity | 0.89 (0.46-1.71) | 1.12 (0.88-1.44) |
| Triglycerides, per mmol/L | 0.98 (0.80-1.20) | 1.00 (0.90-1.11) |
| HDL-C, per mmol/L | 0.69 (0.32-1.50) | **0.42 (0.25-0.70)** |
| LDL-C, per mmol/L | 0.88 (0.64-1.21) | 1.14 (0.94-1.38) |
| Lipoprotein(a), per 10 mmol/L | **1.04 (1.00-1.08)** | 1.02 (1.00-1.04) |
| CRP, per mg/L | **1.03 (1.01-1.06)** | 1.00 (0.98-1.02) |
| Vitamin D, per nmol/L | 0.99 (0.98-1.01) | 1.00 (0.99-1.00) |
| Gamma-glutamyltransferase, per 10 U/L | 1.03 (0.99-1.07) | 1.01 (0.99-1.03) |

The model is adjusted for age, gender, ethnicity, education, Townsend [deprivation index,](http://www.mendeley.com/research/townsend-deprivation-index/) physical activity, duration of diabetes, diabetes-related medication, hemoglobin A1c and all listed risk factors. Abbreviations: ASCVD (atherosclerotic cardiovascular disease); HDL-C (high-density lipoprotein cholesterol); LDL-C (low-density lipoprotein cholesterol); CRP (C-reactive protein).
**BOLDED** items are significant statistically significant (P < 0.05).

**Supplementary Table 6.** Multivariable-adjusted hazard ratios (95% confidence interval) for the association of HbA1c and incident atherosclerotic cardiovascular disease among those with type 2 diabetic microvascular complications

|  | Model 1 | Model 2 |
| --- | --- | --- |
| HbA1c, per 5 mmol/mol | **1.03 (1.00-1.05)** | 1.00 (0.96-1.03) |

Model 1 is a crude model; Model 2 is adjusted for age, gender, ethnicity, education, Townsend [deprivation index,](http://www.mendeley.com/research/townsend-deprivation-index/) physical activity, duration of diabetes, diabetes-related medication, cigarette smoking, hypertension, hyperlipidemia, family history of atherosclerotic cardiovascular disease, central obesity, serum high-density lipoprotein cholesterol, serum triglycerides, serum low-density lipoprotein cholesterol, serum C-reactive protein, serum lipoprotein(a), serum vitamin D, and serum gamma-glutamyltransferase. Abbreviations: HbA1c (hemoglobin A1c).

**BOLDED** items are significant statistically significant (P < 0.05).

**Supplementary Table 7.** Multivariable-adjusted hazard ratios (95% confidence interval) for the association of ASCVD risk factors and incident atherosclerotic cardiovascular disease among those with type 2 diabetic microvascular complications stratified by HbA1c at baseline

| ASCVD risk factors | HbA1c | |  |
| --- | --- | --- | --- |
|  | **≤50.6nmol/mol** | **>50.6nmol/mol** | **P for interaction** |
| Cigarette smoking-Former/Current | 1.13 (0.80-1.58) | 1.18 (0.93-1.51) | 0.587 |
| Hypertension | 0.85 (0.43-1.66) | 1.03 (0.67-1.59) | 0.739 |
| Hyperlipidemia | 1.09 (0.69-1.73) | 1.19 (0.83-1.70) | 0.637 |
| Family history of ASCVD | 1.25 (0.89-1.75) | 1.22 (0.96-1.55) | 0.783 |
| Central Obesity | 1.16 (0.80-1.68) | 1.05 (0.79-1.41) | 0.656 |
| Triglycerides, per mmol/L | 1.00 (0.85-1.19) | 0.96 (0.85-1.07) | 0.816 |
| HDL-C, per mmol/L | 0.49 (0.24-1.02) | **0.52 (0.31-0.87)** | 0.809 |
| LDL-C, per mmol/L | 1.04 (0.80-1.36) | 1.11 (0.92-1.35) | 0.777 |
| Lipoprotein(a), per 10 mmol/L | 1.02 (0.98-1.05) | 1.01 (0.99-1.04) | 0.762 |
| CRP, per mg/L | 0.99 (0.97-1.01) | **1.02 (1.01-1.04)** | **0.009** |
| Vitamin D, per nmol/L | 1.00 (0.99-1.01) | 1.00 (0.99-1.00) | 0.382 |
| Gamma-glutamyltransferase, per 10 U/L | 1.02 (0.99-1.05) | 1.02 (1.00-1.04) | 0.508 |

The model is adjusted for age, ethnicity, education, Townsend [deprivation index,](http://www.mendeley.com/research/townsend-deprivation-index/) physical activity, duration of diabetes, diabetes-related medication, and all listed risk factors. Abbreviations: ASCVD (atherosclerotic cardiovascular disease); HbA1c (hemoglobin A1c); HDL-C (high-density lipoprotein cholesterol); LDL-C (low-density lipoprotein cholesterol); CRP (C-reactive protein).
**BOLDED** items are significant statistically significant (P < 0.05).
